# Cardiovascular Risk Factors in the Antiphospholipid Syndrome

**DOI:** 10.1155/2014/621270

**Published:** 2014-07-13

**Authors:** Felipe Freire da Silva, Roger Abramino Levy, Jozélio Freire de Carvalho

**Affiliations:** ^1^Escola Bahiana de Medicina e Saúde Pública, 40290-000 Salvador, BA, Brazil; ^2^Rheumatology Division, Universidade Estadual do Rio de Janeiro, 20550-900 Rio de Janeiro, RJ, Brazil; ^3^Rheumatology Division, Centro Médico do Hospital Aliança, 41810-080 Salvador, BA, Brazil

## Abstract

A major cause of morbidity and mortality in the context of the antiphospholipid syndrome (APS) is the occurrence of thrombotic events. Besides the pathogenic roles of antiphospholipid antibodies (aPL), other risk factors and medical conditions, which are conditions for traditional risk of an individual without the APS, can coexist in this patient, raising their risk of developing thrombosis. Therefore, the clinical and laboratory investigation of comorbidities known to increase cardiovascular risk in patients with antiphospholipid antibody syndrome is crucial for the adoption of a more complete and effective treatment. Experimental models and clinical studies show evidence of association between APS and premature formation of atherosclerotic plaques. Atherosclerosis has major traditional risk factors: hypertension, diabetes mellitus, obesity, dyslipidemia, smoking, and sedentary lifestyle that may be implicated in vascular involvement in patients with APS. The influence of nontraditional risk factors as hyperhomocysteinemia, increased lipoprotein a, and anti-oxLDL in the development of thromboembolic events in APS patients has been studied in scientific literature. Metabolic syndrome with all its components also has been recently studied in antiphospholipid syndrome and is associated with arterial events.

## 1. Introduction

Antiphospholipid syndrome (APS) is an autoimmune disease characterized by recurrent fetal loss, thrombocytopenia, and a procoagulant state resulting in frames of arterial and venous thrombosis. This clinical presentation occurs in the presence of antibodies that act on membrane phospholipids, such as anticardiolipin (aCL), lupus anticoagulant (LAC), and anti-*β*2-glycoprotein I (anti-*β*2GPI) [1].

A major cause of morbidity and mortality in the context of the APS is the occurrence of thrombotic events, which may affect any arterial or venous vascular bed. Manifestations are common in these patients: deep vein thrombosis, pulmonary thromboembolism, stroke, transient ischemic attack, and coronary artery disease [2].

The possible roles of antiphospholipid (aPL) in the pathogenesis of thrombosis in APS are well established in the scientific literature, including changes in the coagulation cascade, including inhibition of protein molecules C, antithrombin, and annexin, platelet activation and complement, and increased expression of endothelial adhesion molecules [3].

However, other risk factors and/or medical conditions, which are conditions for traditional risk of an individual without the APS, can coexist in this patient, raising their risk of developing thrombosis. Examples of these conditions are obesity, dyslipidemia, hypertension, diseases, diabetes mellitus, smoking, and hyperhomocysteinemia [4].

In view of the clinical manifestations of the antiphospholipid syndrome that often involve serious consequences, such as thrombotic events, it is necessary to know other factors and conditions that may result in a worse outcome. Therefore, the clinical and laboratory investigation of comorbidities known to increase cardiovascular risk in patients with antiphospholipid antibody syndrome is crucial for the adoption of a more complete and effective treatment.

## 2. Experimental Models

The medical literature shows evidence of association between antiphospholipid syndrome and the occurrence of accelerated atherosclerosis. However, the coexistence of traditional risk factors still makes this respect controversial [5].

The correlation between high levels of serum cholesterol and low density lipoprotein (LDL) in the atherosclerotic process is already well defined in the scientific community [6, 7]. However, other factors of chronic vascular injury, of inflammatory and immunological origin, must be considered in addressing the pathogenesis of atherosclerosis [8].

Among the key molecules involved in the atherosclerotic process are the heat shock proteins, the oxidized low density lipoprotein (oxLDL), and *β*2-glycoprotein I (*β*2GPI) [5].

The oxidation of the LDL molecule, including the peroxidation of phospholipids and esters of cholesterol, is one of the most important events early in the disease process and the intimal thickening and consequent narrowing of arterial blood vessels. This endothelial dysfunction could be explained in part by the actions of oxLDL in the recruitment of macrophage/monocytes and T lymphocytes, cytotoxicity to endothelium, and further stimulation of immune responses [8, 9]. And experimental studies in humans have already shown the presence of oxLDL in atherosclerotic plaques [9]. Therefore, inflammatory and immunological conditions requiring chronic vascular injury develop and maintain a cycle of oxidative stress, involving oxLDL, which culminates in a prothrombotic condition [8].

The *β*2GPI molecule (protein bound to phospholipid), in turn, is identified as an antiatherogenic agent, (as demonstrated by George et al. study, where oral tolerance with human and bovine *β*2GPI suppressed early atherosclerotic lesions in LDL- receptor deficient mice) [10] may be associated with the process of formation of atheromatous plaques in APS, while being targeted by the antibodies typical of this condition [5]. In the literature the formation of a stable complex between oxLDL and *β*2GPI, detected in patients with autoimmune disease and/or atherosclerosis [9], and the presence of cross-reactivity between aPL antibodies and anti-oxLDL have also been described, factors that raise suspicion regarding an association between APS and atherosclerosis [5].

Additionally,* in vitro* studies indicated an increase in the uptake of oxLDL complex/*β*2GPI by macrophages upon exposure to *β*2GPI antibodies IgG, giving a proatherogenic role of anti-*β*2GPI in APS [9].

Experimental studies have also demonstrated an association between aPL and atheromatosis from immunization of groups of mice with deficiency of LDL receptor. In one experiment, three groups maintained on a standard diet were immunized with *β*2GPI or ovalbumin or have not been immunized. Meanwhile, three other groups of mice were immunized in the same way, and, however, underwent an atherogenic diet. As a result, all animals immunized with *β*2GPI develop *β*2GPI antibodies and subsequent acceleration of atherosclerosis compared to those immunized with ovalbumin and nonimmunized [10]. Similarly, mice with deficiency of apolipoprotein E that were immunized with *β*2GPI develop atherosclerosis more in Afek et al. [11].

In another experiment, it was evident that the anticardiolipin antibody (aCL) increased atherogenesis in mice with deficiency of the LDL receptor, suggesting the participation of this antibody in the development of atherosclerosis in patients with APS [12].

## 3. Clinical Evidences

In order to base the suspected association between the APS and premature atherosclerosis, several clinical studies have addressed patients with persistence of aPL from the use of noninvasive exams aimed at early detection of atherosclerosis.

The intima-media thickness (IMT) of peripheral arteries, being important, can be estimated relatively simply and inexpensively by B-mode ultrasonography. This method has been used, therefore, in assessing the extent of the atherosclerotic process, predicting cardiovascular risk in the study subjects [13].

Thus, a prospective study involving 58 patients with APS and 58 controls assessed the EMI, vascular stiffness, and presence of plaques in the carotid and femoral from Doppler ultrasound and found as a result statistically significant differences between groups. This work demonstrated, however, a predisposition to atherosclerosis in patients with primary and secondary APS, and there was no correlation between the LES and the formation of atherosclerotic plaques and independence between markers atheroma with cardiovascular risk factors and inflammation [14].

Additionally, a recent review selected scientific papers that investigated the carotid artery in patients with APS [15]. As a result, three of the included studies that, overall, involved 58 patients with APS and 79 controls showed higher IMT in groups with primary APS compared with the control groups [16–18]. Two other studies have found no statistically significant difference [19, 20].

In the study of Bilora et al., atherosclerosis was evident from the use of ultrasonography: in the carotid arteries in 35.6% of patients and 35.0% of controls, in the arteries of the lower limb in 26.7% of patients and 20.0% of controls, and in the abdominal aorta in 20.0% of patients and 17.5% of controls. It also detected a total number of plates 60/45 in the experimental sample and 54/40 in the control group [20].

High levels of EMI and reduced luminal volume were found in patients with APS in Charakida et al. study, which raised the possibility of association between intima-media thickness in APS and stroke [21].

Using the evaluation of magnetic resonance imaging, other scientific publication that covered 27 APS patients and 81 controls, both that underwent cardiac magnetic resonance imaging with gadolinium contrast, showed that approximately 30% of APS patients had silent coronary artery disease and 11.1% of them showed typical patterns of myocardial infarction. This work has not identified association between coronary artery disease in APS and classical cardiovascular risk factors [22].

The evaluation of patients with primary APS from the use of positron emission tomography (PET) revealed the presence of endothelial dysfunction that may contribute to the acceleration of atherosclerosis in APS [5, 23].

The use of contrast echocardiography and nuclear imaging in patients with APS showed higher levels of defects in myocardial perfusion in the study of Espinola-Zavaleta et al., reinforcing the association with atherosclerotic manifestations in this group of patients with autoimmune disease [24].

One vascular manifestation also described in the literature as a complication of APS is the occurrence of abdominal aortic aneurysms, which raises questions about the need for screening this condition and imposes danger in cases of rupture and subsequent hemorrhage [25].

A simple clinical method, noninvasive and easily applied in the investigation of peripheral vascular disease, is the ankle brachial index (ABI). In this regard, Barón et al. demonstrated through its clinical study abnormal ABI in patients with primary APS [26].

Finally, the use of transcranial ultrasound also demonstrated possible association with AT subclinical FAS by enhancing cerebral blood flow abnormalities in patients with primary antiphospholipid syndrome without neurological symptoms [5, 27].

## 4. Traditional Risk Factors

Atherosclerosis has major traditional risk factors: hypertension, diabetes mellitus, obesity, dyslipidemia, smoking, and sedentary lifestyle. These comorbidities may act on inflammatory mechanisms and lipid metabolism contributing to the process of vascular injury, which therefore will trigger and propagate the formation of atherosclerotic plaques. It is suspected that this process may be implicated in vascular involvement in patients with antiphospholipid syndrome [8].

A recent cross-sectional study with 39 patients with primary APS found prevalence of traditional risk factors: 46.2% for hypertension, 12.8% for diabetes mellitus, 28.2% for hypercholesterolemia and high LDL, 15.4% of hypertriglyceridemia, 84.6% for low HDL, 28.2% of family history, 56.4% of sedentary, 15.4% with a history of smoking, and 12.8% of current smokers. In this study, we compared groups of patients with primary antiphospholipid syndrome versus secondary APS; the only risk factor that showed a significant difference between the two samples was the HDL-c, more frequent in primary APS [28].

Souza et al. showed hypertension as traditional cardiovascular risk factor most strongly associated with arterial thrombotic process that occurs in the APS. This study evaluated the presence of traditional risk factors for coronary artery disease (CAD), along with other factors in 38 patients with primary APS and 30 controls, investigating possible association with arterial thrombosis. The average number of risk factors was higher in the experimental group, and, notably, hypertension was the only independently associated with arterial thrombosis. Regarding the lipid profile, patients exhibited higher levels of LDL and triglycerides and lower levels of HDL compared with the control group [29].

The cross-sectional study of Erkan et al., in turn, involved 77 patients with APS and 56 asymptomatic aPL and, after consideration of traditional risk factors, found no significant difference between groups. However, hypertension and smoking were associated with arterial thrombotic events. The arterial involvement in this work was also related to the combination of cardiovascular risk factors [30].

In this same line of research, one Chinese work retrospectively analyzed 61 patients with primary APS and also showed hypertension as an independent risk factor for the development of arterial thrombosis [31].

Girón-González et al. analyzed prospectively 404 patients with aPL, divided into 2 groups: those with primary or secondary APS and those with asymptomatic aPL. While the presence of past surgery and immobilization were associated with the occurrence of venous thrombosis, hypertension again and this time also dyslipidemia were more prevalent in patients with arterial thrombosis. In addition, half of the patients with APS had coincident cardiovascular risk factor, in contrast to the control group [32].

The study of Bilora et al., which included 45 patients with APS and 40 with secondary deep venous thrombosis despite having found a higher frequency of hypertension and diabetes mellitus in the group with APS, did not show statistically significant differences [20].

Medina et al. and Der et al. demonstrated that the cardiovascular risk factors, obesity, hypertension, and von Will brand factor were more frequently found in patients with APS and, however, showed no relationship with intima-media thickness [15–17].

In the medical literature also some case reports on the association between APS and diabetes mellitus are described, generally showing an angiopathy frame. However, two recent reviews have evaluated the endocrine manifestations of APS and found inconsistent data with this relationship. One of the reviews highlighted studies which described increases in the frequency of aPL antibodies in the serum of diabetic patients implying macroangiopathic complications, besides the finding of aCL IgG antibodies in the serum of first-degree relatives of diabetic patients. On the other hand, another literature review highlighted the need for further studies to clarify the relationship between aPL and diabetes mellitus, one time found no consistent data on the prevalence and significance of aPL in diabetes mellitus [33, 34].

Smoking is a major risk factor for atherosclerosis by inducing vascular damage that hinders the endothelium-dependent vasodilation, probably through its various toxic compounds such as carbon monoxide and nicotine [35]. Smoking cessation, therefore, tends to reduce the risk of thromboembolism [36], preventing the occurrence of serious cardiovascular events such as acute myocardial infarction (AMI) and stroke; complications are very prevalent in patients with APS.

A recent study associated the occurrence of seizures in patients with APS (present in 10.2% of patients with primary APS study) with current smoking and stroke, establishing itself as an independent factor, calling attention therefore to the importance of smoking cessation in these patients, in addition to the same need to control other cardiovascular risk factors that may be present [37].

Obesity is a factor which has contributed much to increased morbidity and mortality in the general population, especially for being involved with vascular complications. This concern with the nutritional status of the patient is, in turn, even more valued in the course of a pregnancy due to the risk of maternal and fetal complications that obesity can cause. Considering such losses, this is one of the main risk factors that must be fought in individuals with APS [38, 39]. A study by Caldas et al. showed worse prognosis in obese patients with primary APS compared with those who were not obese. In this transversal work, 50 patients with primary APS were divided into 2 groups: APS obese (BMI ≥ 30 kg/m^2^) and APS nonobese (BMI < 30 kg/m^2^). The obese group had a higher frequency of pulmonary embolism (PE) and obstetric events [38].

Klack and Carvalho evaluated the efficacy of nutritional intervention in the treatment of overweight in 40 APS patients treated at least after 2 months and had positive results in the adequacy of body weight in these patients, indicating the nutritional intervention as a first choice in obese individuals with APS [39].

## 5. Nontraditional Risk Factor

The influence of traditional cardiovascular risk factors in the development of thromboembolic events in patients with APS is relatively well explored in the literature. However, it is also necessary to highlight the role of nontraditional risk factors in this process, such as hyperhomocysteinemia and lipoprotein a [40].

Homocysteine is the amino acid product of the demethylation of methionine which occurs intracellularly. High levels of this molecule have been associated with an increased frequency of vascular events such as myocardial infarction, stroke, and peripheral arterial disease [41].

A recent cross-sectional study involving 27 patients with primary APS found a percentage of 22% of hyperhomocysteinemia. However, when comparing this sample with individuals who had normal levels of homocysteine, there were no significant clinical and laboratory changes [40].

Another study that included 38 women with primary APS found higher levels of homocysteine in patients with arterial thrombosis than in controls, although only hypertension was independently associated with arterial thrombosis in this study [29].

A lipoprotein is a circulating lipid particle recognized as an independent risk factor for the formation of atherosclerotic plaques and subsequent cardiovascular events (Carvalho and Liming, 2009 [42]).

A cross-sectional study that selected 46 patients with primary APS found a frequency of 43.5% of the patients with elevated lipoprotein; however, there was no significant association with clinical and laboratory changes.

As already mentioned in this paper, oxLDL molecule forms a complex with *β*2GPI which can be detected in the serum of patients with APS. Laczik et al. evaluated the effect of oxLDL in the pathogenesis of APS which leads to acceleration of the atherosclerotic process and concluded based on their clinical study that this molecule probably contributes to the perpetuation of the immune process in APS through the production of Th1-type cytokines and lymphocyte proliferation [43]. Similarly, after a clinical evaluation of the association between oxLDL and anti-oxLDL with primary APS, Becarevic et al. characterized these molecules as additional risk factors for the occurrence of thrombotic events in APS [44].

## 6. Metabolic Syndrome and Adipocytokines

Metabolic syndrome (MS) is a condition in which an individual is present in at least 3 of the following 5 criteria: waist circumference (>102 cm in men and >88 cm for women), hypertriglyceridemia (≥150 mg/dL), low HDL (<40 for men and <50 for women), blood pressure ≥ 130 × 85 mm Hg, and blood glucose ≥ 110 mg/dL [45]. The diagnosis of MS requires, therefore, a high cardiovascular risk, which should be investigated and fought mainly in patients with chronic rheumatic diseases, which are already subject to increased risk of thromboembolic events by own mechanisms of disease or by treatment with corticosteroids. Studies indicate higher frequency of MS in patients with rheumatic diseases than in the general population [46].

Rodrigues et al. evaluated 71 patients with primary APS and 73 controls to evaluate clinical findings of MS in individuals with FASD. As a result, the prevalence of MS in the experimental group was 33.8%. Subgroup analysis of APS with or without associated SM showed higher frequency of arterial events, angina, and LAC positive in the first group. In multivariate analysis, only the SM was independently associated with arterial events in primary APS [47, 48].

The white adipose tissue is considered by some authors as a possible endocrine organ because of some of its actions as metabolic regulation of bodily functions such as thermogenesis, feeding, and lipid and glucose metabolism and cardiovascular function. Obesity, therefore, to generate hypertrophy and hyperplasia of adipocytes, elevates levels of adipocytokines, which may lead to reduced insulin sensitivity, increased contractility, and vascular inflammation [49].

A study by Rodrigues et al. evaluated 56 patients with primary APS and 72 controls finding evidence of the association between adipocytokines and low-grade inflammation, insulin resistance, and metabolic syndrome in patients with primary APS. The leptin was found at high levels in this work, being associated with increased waist circumference and body mass index (BMI). Although assigning a role and connection with atherogenic cardiovascular events, this study showed no significant difference between high levels of this hormone and the occurrence of arterial thrombosis [47, 48].

## Key Messages

We have the following points.

- Cardiovascular disease is a manifestation of the antiphospholipid syndrome.
- Cardiovascular disease is associated with antiphospholipid antibodies, traditional risk factors (hypertension, obesity, dyslipidemia, etc.), and nontraditional risk factors (hyperhomocysteinemia, increased lipoprotein a, etc.).
- Metabolic syndrome with all its components has been recently studied in antiphospholipid syndrome and is associated with arterial events.
